# Behavioral Sequence Analysis Reveals a Novel Role for ß2* Nicotinic Receptors in Exploration

**DOI:** 10.1371/journal.pcbi.1000229

**Published:** 2008-11-21

**Authors:** Nicolas Maubourguet, Annick Lesne, Jean-Pierre Changeux, Uwe Maskos, Philippe Faure

**Affiliations:** 1 Unité Neurobiologie Intégrative des Systèmes Cholinergiques, Institut Pasteur, Paris, France; 2 CNRS, URA 2182, Paris, France; 3 Institut des Hautes Etudes Scientifiques, Bures-sur-Yvette, France; University College London, United Kingdom

## Abstract

Nicotinic acetylcholine receptors (nAChRs) are widely expressed throughout the central nervous system and modulate neuronal function in most mammalian brain structures. The contribution of defined nAChR subunits to a specific behavior is thus difficult to assess. Mice deleted for ß2-containing nAChRs (ß2−/−) have been shown to be hyperactive in an open-field paradigm, without determining the origin of this hyperactivity. We here develop a quantitative description of mouse behavior in the open field based upon first order Markov and variable length Markov chain analysis focusing on the time-organized sequence that behaviors are composed of. This description reveals that this hyperactivity is the consequence of the absence of specific inactive states or “stops”. These stops are associated with a scanning of the environment in wild-type mice (WT), and they affect the way that animals organize their sequence of behaviors when compared with stops without scanning. They characterize a specific “decision moment” that is reduced in ß2−/− mutant mice, suggesting an important role of ß2-nAChRs in the strategy used by animals to explore an environment and collect information in order to organize their behavior. This integrated analysis of the displacement of an animal in a simple environment offers new insights, specifically into the contribution of nAChRs to higher brain functions and more generally into the principles that organize sequences of behaviors in animals.

## Author Summary

Understanding mechanisms underlying complex behaviors and the abnormalities that accompany most neuropathologies is a current challenge in biomedical research. A number of approaches is primarily based on the identification of genes and their associated molecular pathways implicated in complex motor or cognitive pathologies. However, optimal use of the large body of genetic, molecular, electro-physiological, and imaging data is hampered by the practical and theoretical limitations of currently available behavioral analysis methods. Complex behaviors consist of a finite number of actions combined in a variety of spatial and temporal patterns. In this paper we develop a sequential analysis of mouse displacement in an open-field paradigm and demonstrate that a description based on a Markov model can be used to describe quantitatively patterns of behaviors and to detect changes in the way that animals organize their displacement, especially in mice lacking nicotinic acetylcholine receptor subunits. This paper would be of broad interest not only to those concerned with this particular mice model but also generally to those interested in modeling complex behavior traits in mice.

## Introduction

nAChRs are well-characterized transmembrane allosteric oligomers composed of five identical (homopentamers) or different (heteropentamers) subunits [1]. Nine different subunits are widely expressed in the mammalian brain, modulating neurotransmitter release, neuronal excitability and activity dependent plasticity in most, if not all, mammalian brain structures [2],[3]. The elementary mechanisms of nAChRs functions are investigated in great details, yet important issues relevant for the role of nAChRs at the higher level, have received less attention. The need to fill this gap is reinforced by nAChR participation in a diverse array of neuropathologies, including Alzheimer's disease, Parkinson's disease, schizophrenia, epilepsy and Attention-deficit hyperactivity disorder. The complex nature of all these disorders underlines the nicotinic influences over neuronal circuits involved in attention, motivation and cognition [2],[3].

The issue then becomes how to tackle this problem in mouse models that allow pharmacological and genetic manipulations, but for which “psychological” processes must be inferred from observable behaviors. Mice deleted for ß2-subunit containing nAChR (ß2−/−) have been the first nicotinic receptor mutant to be characterized, and found to exhibit more rigid behavior and less behavioral flexibility than wild-type (WT) animals [4]. Overall, these experiments suggest that ß2−/− mice reduce the time allocated to explore a novel environment [4],[5]. Lentiviral reexpression techniques indicate that this phenotype is linked to the expression of ß2*-nAChRs in the ventral tegmental area [6],[7] and in the Substantia Nigra [8].

ß2−/− mice were shown to be hyperactive in an open-field paradigm, with a reduced movement at low speed, and consequently an increased movement at high speed. Hyperactivity in an open field is often used as a general and non-specific term characterizing experimental conditions where animals show either an increased amount of displacement and related locomotor behaviors, or changes in the frequency of specific motor acts [9]. Increased locomotor activity in an open field can reflect different processing and alterations in the organization of behavior [9]. A complete description of hyperactivity then requires to study duration and temporal patterning (i.e. the sequence) of behavioral acts. In this paper, we address the problem of tracing, by analyzing temporal organization of movement, mouse cognitive and/or decision making behavior that can account for mouse hyperactivity in the open-field.

Open-field behaviors have been used to study forced exploration of a new environment. It has been shown that it involves both exploratory and stress/fear components [10]–[13]. Furthermore, kinematic features based on instantaneous speed and location have been used to demonstrate that rat and mouse trajectories are far from random [14],[15], and that animals can stop more frequently in specific locations of the field that structure their trajectory [16],[17]. Here, we focus on the analysis of the behavioral sequence, namely the time-organized sequence of patterns that composes the behavior. Considering a sequence of acts, a question would be whether information contained in the structure of this sequence and the presence of specific associations between acts reflects decision-making behavior and can be used to assess alterations of this process.

We developed further the method already successfully applied to detect modifications of locomotor behavior caused by mutations in ß2−/− mice [4],[6], or in goldfish [18]. The principle of the method is to decompose animal trajectories into a combination of discrete units extracted by applying a threshold to continuous variables. We show that the use of a variable-length Markov model [19] to analyze the sequence of symbols allows to unravel significant alterations in the way ß2 mutant mice organize their behavior, and use “stops” to explore their environment.

## Results

### Hyperactive Behaviors in ß2−/− Mice Reflects a Decrease in the Duration of Inactive States

**Figure 1:**
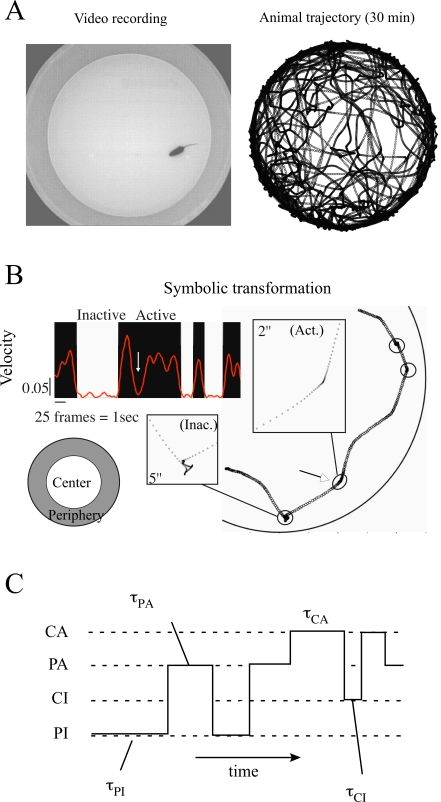
Principle of decomposition of behavior into subunits. (A) Mouse in an open field (1 meter diameter), and two-dimensional trajectory of 30 minutes duration. Position of the animal is here digitized at 25 frames per second. (B) Transformation of continuous variables, velocity and position, into binary symbols. A velocity threshold was set to differentiate inactivity (I - White) and activity (A - Black) periods. Sample of trajectories with two enlarged periods corresponding to an inactivity period and to a velocity decrease following a change in direction (marked by an arrow in the velocity graph) and not identified as an inactivity period. Furthermore, the arena was divided into two concentric zones, P (periphery, shaded) and C (center), the radius of the latter being equal to 0.65. (C) Symbolic sequence analysis: Combining symbols leads to the definition of four states PI | PA | CI | CA. The trajectory is then represented by a sequence of symbols (marked by steps) and associated residence times (τ).

Both WT and ß2−/− mice were active in the open-field. They exhibited movements along the wall, sequences of trajectories in the middle of the field (Figure 1A), and alternation between locomotor progression and periods of slow movements. This allowed us to describe locomotor activity in terms of a sequence of four states {PI, PA, CI, CA} (Figure 1B and 1C).

**Figure 2:**
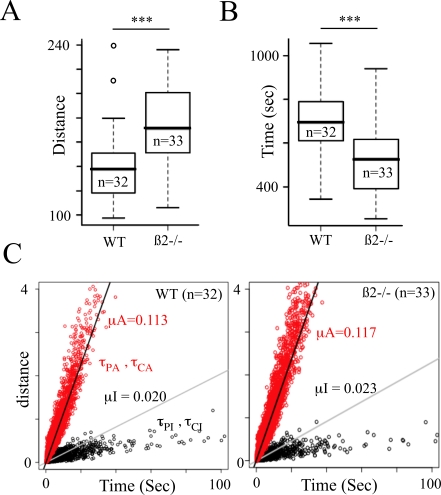
Relation between duration of state and traveled distance. (A) Boxplot of the total traveled distance and (B) time spent in inactive state during a 30 min session in the open-field respectively for wild-type (WT, n = 32) and mutant mice (ß2−/−, n = 33). (C) Relation between the times spent in a given state (PA and CA in red, PI and CI in black) and the distance traveled during this time. Best linear fits were indicated for active and inactive states with the respective slope (µ). Number of stars indicates the statistical level of significance (- p>0.05, * p<0.05, ** p<0.01, *** p<0.001).

ß2−/− mice have been shown to be hyperactive in the open-field (Granon et al 2003, Avale et al, 2008), with a distance traveled during 30 min being 1.25 times longer in KO compared to WT mice (Figure 2A, Δ = 34.57 m). This hyperactivity was reflected in the time spent in an inactive or active state with a decreased time in the inactive state in mutant mice (Figure 2B). The relation between the distance traveled and the duration of the different states were however not different in the two strains. For both strains, the distance traveled during active or inactive states was different, but both exhibited a linear relationship with the duration of a given event (μ = 0.113 and 0.117 in active phase for wt and ß2−/− mice, and μ = 0.02 and 0.023 in inactive phase). These relationships tended to break down for long events, but were not different in WT (Figure 1C, left) and in ß2−/− mice (Figure 1C, right). The distance traveled was then roughly reflected in the time spent in inactive or active states. These results suggest that higher locomotor activity in ß2−/− mice is not due to a modification of the velocity distribution (either in the active or inactive phase), but rather to a significant change in the organization of the behavior.

### First-Order Markov Description of the Animal Trajectory Is Modified in ß2−/− Mice

**Figure 3:**
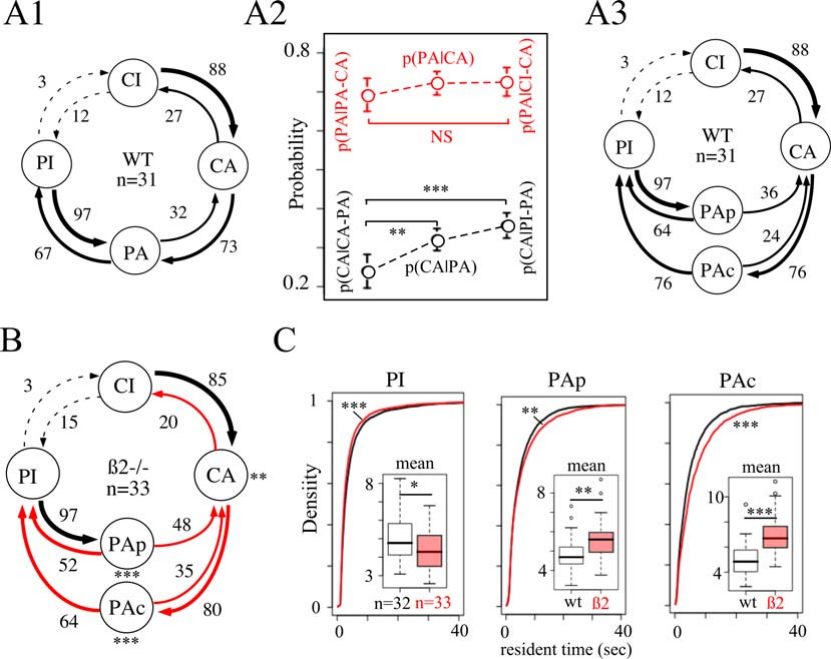
First-order matrix of transition. (A) Flow diagram: (A1) Transition matrix between the four states can be used to build a flow diagram, where conditional probabilities of transition between states are indicated by number (percentage) and by the thickness of the connecting arrows. Transitions from PI to CA and CI to PA are almost never observed (p<1/1000) and then are not represented in the flow diagram. (A2) Conditional probability of transition from PA to CA depends on previous state. Comparison of P(X|YZ) and P(X|Y) for X = PA, Y = CA (red points) and Z = PA (left) or CI (right) indicates no significant difference (NS). For X = CA, Y = PA (black points) and Z = PA (left) or PI (right) a significant difference appear. (A3) First-order Markov description of the sequence with a distinction between Pap and PAc (see text). (B) First-order Markov description of ß2−/− sequence. Red connecting arrows indicate probabilities of transition that are statistically modified when compared with WT. (C) Comparison of the distributions of time spent within PI, PAp and PAc states respectively (from left to right). Inset: Boxplot of the mean duration of the indicated state. Number of stars indicates statistical level of significance (- p>0.05, * p<0.05, ** p<0.01, *** p<0.001).

A change in the time spent in inactive states does not give any insight into the modification of the temporal structure of behaviors. Analysis of transition frequencies and conditional probabilities between different states of the animal were then carried out (Figure 3A1). Using only four states {PI, PA, CI, CA} did not allow to build a first-order Markov description of the sequence of states. Indeed, when checking for all possible combinations of states X, Y, Z whether P(X|YZ) = P(X|Y) was satisfied, revealed that the probability of states X after Y = PA did not depend only on the present state PA, but also on the previous one Z (Figure 3A2). In order to obtain a first order Markov dynamics, PA symbols had to be differentiated into peripheral movement that follows central movement (CA), and peripheral movement that follows inactivity in the periphery (PI). They will be designated by the symbols PAc and PAp, respectively. Using the five symbols {PAc, PAp, PI, CA, CI} allowed to describe open-field activity by a first-order process (Figure 3A3). This implies that, with such a state description, the animal movement depends only on the preceding state, suggesting a very local organization of decision-making. The same description could be applied to ß2−/− mice. However, in mutants, the percentage of transitions from periphery to center (PA → CA) was enhanced, while the “stops in the center” transitions (CA → CI) were reduced (Figure 3B).

Stationarity has been tested by comparing transition probabilities obtained during the first and the second 15 minutes of the experiment. We observed (i) a slight modification of (PI → PA) probability of transition (it decreases from 97.7% to 95.5%, and from 98.0% to 96.0% in WT and ß2−/− respectively), and (ii) an increase of (CA → CI) transition with time (from 22.3% to 32.2 and from 13.9% to 25.3 in WT and ß2−/− mice respectively). This last modification indicates that animals have a higher tendency to stop at the center in the second part of the experiment. This increase is similar in WT and in ß2−/− mice.

Distributions of residence times were also modified in ß2−/− mice (Figure 3C). Comparison of the mean of residence times in individual using the Wilcoxon test indicated that PI , PAc and PAp residence times were significantly modified in ß2−/− mice. PI average duration was reduced 13% (Δ mean = 0.58 sec, p = 0.028), while PAp and PAc average duration were increased 15.2 and 35.3% (Δ mean = 0.72, p = 0.0017 and 1.66 sec p = 1.7e-6), respectively. Mean of CI or CA states were not statistically modified, despite an apparent difference in the distribution of CI (not shown).

In the state sequence, CA is preceded either by PAp, PAc or CI. In WT, there was no significant difference between time distributions of CA, depending on the preceding state (Wilcoxon test). In contrast, CA resident time was increased after a CI when compared with PI preceding a PAp or a PAc (mean = 3.09 against 2.7 and 2.8 sec, Wilcoxon test, p<0.001 in both cases). Similar dependencies on preceding state were observed for PI state duration. Mean duration varied significantly (mean = 4.01, 5.16 and 4.11 sec, Wilcoxon test, p<0.001 in pair comparison) after CI, PAc or PAp, respectively (mean = 4.01, 5.16 and 4.11 sec, Wilcoxon test, p<0.001 in all pair comparisons). Similar properties were observed in ß2−/− mice (mean = 3.08, 4.32 and 4.08 sec, Wilcoxon test, p<0.001 in all pair comparisons).

### Elements Explaining Hyperactivity

Deletion of the ß2-subunit gene affected both the residence time distribution and the transition matrix. To identify more specifically the locus of the behavioral sequence where the mutation effect takes place, we used a modeling strategy (see Methods).

**Figure 4:**
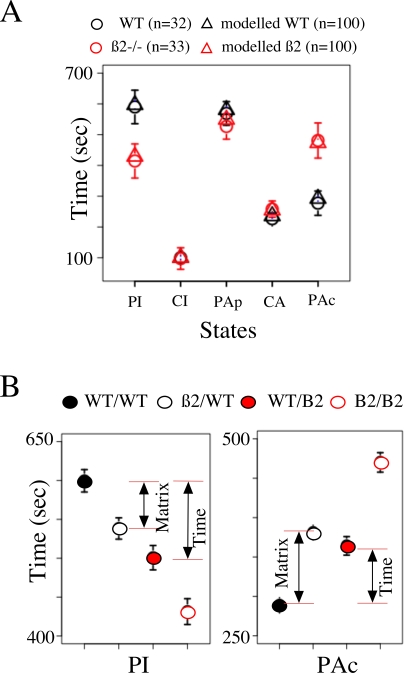
Simulation of the sequence. (A) Comparison between the total time spent in PI, CI, PAp, CA and PAc states (from left to right) during a 30 min session in the open-field, for WT (black circle) and ß2−/− (red circle) and with the simulation obtained from WT first-order transition matrix and residence time distributions (black triangles) and with the simulation obtained from ß2−/− first-order transition matrix and residence time distributions (red triangles). Note that distributions of experimental and simulated data fit perfectly meaning that the simulations reproduce the dynamics as regards the average time spent in each state. (B) Simulated time spent in PI (left) and PAc (right) obtained by combining transition matrices and distributions of state durations (see text). WT/WT, ß2/WT, WT/ß2 and ß2/ß2 indicate that sequences are simulated using WT or ß2−/− matrices of transition (before /) and WT or ß2−/− state duration distributions (after /). (e.g. WT/ß2 indicates simulation with WT matrix of transition and ß2−/− residence time distribution). “Matrix” and “Time” indicate that the discrepancy originates from the effect of changing the transition matrix and the residence time distribution, respectively.

We first checked the validity of the simulation (see also Text S1 and Figure S1 and Figure S2) and that the numbers of occurrences of each of the five states in 30 min experiment agreed well in both WT and ß2−/− mice with numbers obtained with simulated data when the respective matrix of transition and residence times were used. Accordingly, the total traveled distance being almost linearly (Figure 2C) related to the total time spent in each of the five states, it was also well-reproduced using simulation (Figure 4A). We also tested the impact of non-stationarity and resident time sequence dependency (see also Text S1 and Figure S1) on the simulation.

To further dissect the respective contribution of the transition matrix and of the residence time distributions, we modeled data based on: (i) transition matrix of WT and residence time distribution of WT (labeled WT/WT), (ii) transition matrix of ß2−/− and residence time distribution of WT (ß2/WT), (iii) transition matrix of WT and residence time distribution of ß2 (WT/ß2), and (iv) transition matrix of ß2−/− and residence time of ß2−/− (ß2/ß2), and we compared the time spent in PI and in PAc (Figure 4B) for the various model configurations. Convolving matrix and residence time distribution demonstrated that none of them fully explained modifications of the time spent in a given state and consequently the “hyperactivity profile”. Transition probabilities and residence time distribution explained individually no more than 56% of the total difference observed between WT and ß2−/−, while their sum effect explained 95 and 92% of the total mean difference observed between WT and ß2−/−. In terms of quantification this suggested that both matrices and distributions of residence time should be used.

**Figure 5:**
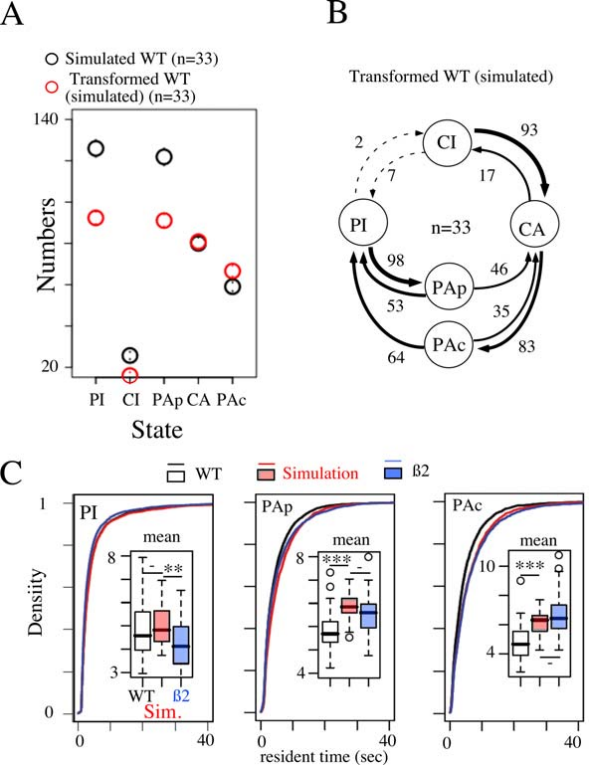
Transformation of WT into ß2−/− profile. (A) Comparison between the number of PI, CI, PAP, CA and PAc states (from left to right) simulated using WT first-order transition matrix and residence time distributions (black circles) and after a transformation consisting in removing a fixed percentage of inactivity (red circles - see Method section and text for the principle of transformation). Note that distributions of experimental and simulated data fit perfectly (see Figure 4A for comparison). (B) First-order Markov description of transformed WT sequence of behaviors. The matrix is similar to those obtained in ß2 KO mice (see Figure 3B). (C) Comparison of the distributions of the time spent within PI, PAp and PAc states respectively (from left to right) for WT (Black, experimental data), simulation (Red) and ß2 (Blue, experimental data). Inset: Boxplot of the mean duration of the indicated state. Number of stars indicates the statistical level of significance (Wilcoxon test, - p>0.05, * p<0.05, ** p<0.01, *** p<0.001).

A final question was whether a single modification of a WT sequence property could reproduce most of the ß2−/− phenotype. The observed behavioral changes between WT and ß2−/− are open to a variety of interpretations. One of them is that ß2−/− specifically reduce some stops. The main advantage of such hypothesis is that modification of only one element (decreased number of stop) accounts for matrix and residence time difference between WT and ß2−/− mice. A simple simulation (see Methods, “stop reduction” model) revealed that removing 30% of stops in WT sequences reproduced well the number of occurrences of each of the five states (Figure 5A), matrices (Figure 5B), and residence time distributions (Figure 5C). More precisely, PI was not changed, which means that the model does not explain the decrease observed in ß2−/− mice. However, Pap and Pac increased to a level compatible with resident time observed in ß2−/− mice (Δ mean = 0.27 sec, Wilcoxon test, p = 0.09 and Δ mean = 0.43 sec, Wilcoxon test, p = 0.49 for Pap and Pac respectively). Such modeling identified the “stop” as an element that could explain differences between WT and ß2−/. We then focused our analysis on this particular moment.

### Ethological Analysis of Inactivity

**Figure 6:**
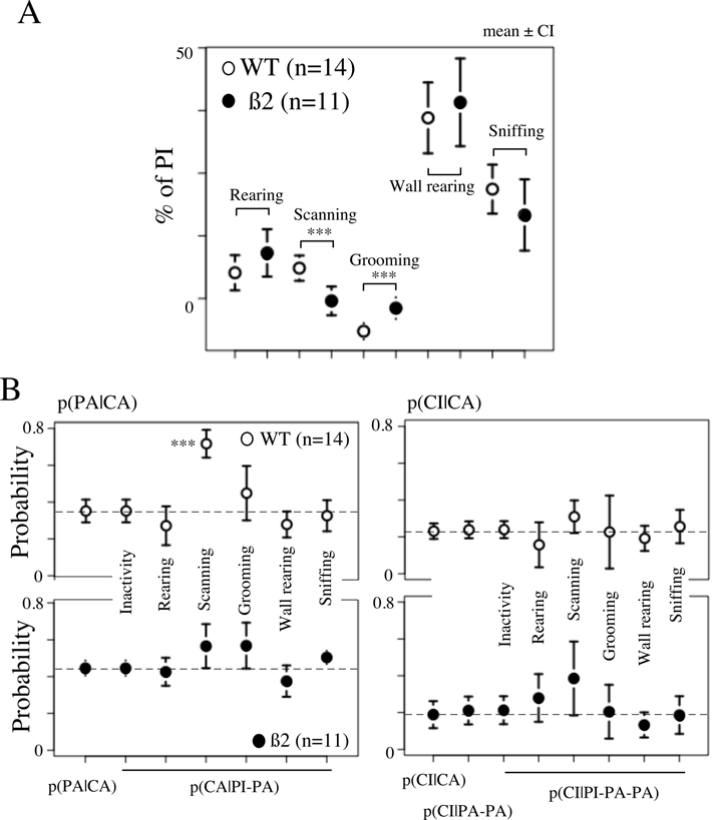
Ethological analysis of inactivity state. (A) Ethogram quantifying activity of the mice during the inactivity state. Comparison of the percentage of rearing, scanning, grooming, wall rearing and sniffing in PI behaviors (see Methods) during a 30 min session in the open-field, in WT (empty circles) and in ß2−/− mice (filled black circles). Number of stars indicates the statistical level of significance (- p>0.05, * p<0.05, ** p<0.01, *** p<0.001). (B) Modification of the probability of the next state depending on activity during a PI. (Left) Modification of P(PA|CA) (indicated by first left point and dashed lines) knowing preceding state i.e Undifferentiated PI, Rearing, Scanning, Grooming, Wall rearing, Sniffing (from left to right), for WT (n = 14, above, white circle) and ß2−/− mice (n = 11, black circle, below). Note that probability of CA is only modified when the mouse performs a scanning (***, p<0.001). (Right) Same presentation for P(CI|CA). Note that this probability is not modified by previous states P(CI|CA) = P(CI|PA-CA) = P(CI|PI-PA-CA), nor by activity performed during a PI (Rearing, Scanning, Grooming, Wall rearing, Sniffing, (from left to right)).

Finite-state systems deriving from the discrete analysis of a continuous movement necessarily coarsen the fine structure of that movement. What has been, so far, identified as inactivity in this paper, is a mode of motion close to a complete stop of the animal. During this period of inactivity the mouse can however make a variety of movements. The animal can progress forward slowly (with a small but constant speed), freeze, perform a number of action patterns (i.e., grooming, rearing, scratching, etc), or orienting movements (head scanning, sniffing, etc). In order to be able to differentiate some of these patterns, we have simultaneously recorded the position of the animal and digitized video images (25 frames/second). These images have been used as the input for fine off-line movement analysis (Figure 6A). Visual analysis of video images allowed us to distinguish periods with rearing and head scanning movements, from periods with only reorientation or no change in orientation. Five classes of inactivity periods were have been distinguished. They corresponded to rearing, scanning, grooming, border rearing and sniffing (see Methods). Stops at the periphery of the open-field were differently distributed in WT (n = 14) and β2−/− (n = 11) mice (Figure 6B). The numbers of rearing, wall rearing, and sniffing were not affected and were similar in both strains (Δ = 3.18, Wilcoxon test, p = 0.32; Δ = 0.4, Wilcoxon test, p = 0.80; Δ = 6.18, Wilcoxon test, p = 0.12, respectively)). Grooming patterns were increased (Δ = 4.1, Wilcoxon test, p = 0.003), whereas scanning was decreased (Δ = 6.9, Wilcoxon test, p = 0.0008) in mutant mice. Scanning behavior being related to the “exploration” of, or the information update about, the environment, differences observed in scanning could therefore have a consequence on the sequence of behaviors.

### Alternative Scanning Choices in ß2−/− Mice

New information obtained by the splitting of PI into five subtypes identified by the dominant behavioral acts, i.e. rearing, scanning, etc., can challenge the description of the sequences in two ways. First, the knowledge of the animal acts during a PI state can modify the probabilities of consecutive states without modifying the first-order Markov description. Second, new information about PI can modify not only the conditional probabilities but also the order of the Markov description, thus requiring a more complex description of the process.

The conditional probability of transition from PA to CA was modified by the knowledge of the behavioral act performed during stops preceding PA (Figure 6B, left (top), ANOVA, F(6,91) = 13.4, p = 8e-11). More specifically, P(CA|PA) = P(CA|PI-PA), when no further indication is given on PI, but the probability of transition was greatly enhanced when the animal performed scanning. That is, P(CA|PA)<P(CA|PIsc-PA) if PIsc was a scanning behavior (Δ = 0.36, test p = 1.5 e-08). These results showed that after scanning an animal tended to engage more frequently in a transition to the center of the arena than after a stop paired with a different activity. Probability to stop at the center of the arena was however not modified by the activity of mice during a PI (Figure 6B, left (bottom), ANOVA, F(7,104) = 0.91, p = 0.49). In ß2−/− mice, the modification of probability after scanning disappeared, that is, the first order model was not modified by knowledge of the behavioral act occurring during a PI (Figure 6B, right).

Providing new information about the PI state modified the Markov order of the description. We therefore switched to Variable Length Markov Chain modeling (see Methods).

### Structural Description of the Decision Tree

If we consider two main populations of stops, i.e. scanning and no-scanning, a tree representation of the influence of the past behavior, i.e “the context”, on a given decision can be built. For this purpose, the sequence of symbols was fitted using a Variable Length Markov Chain model (VLMC, see Methods). Animal trajectories were described using six symbols CI, CA, PAp, PAc, PInsc and PIsc, the two last states coding for stop at the periphery without or with scanning, respectively. Sequences from different animals were concatenated for VLMC analysis.

**Figure 7:**
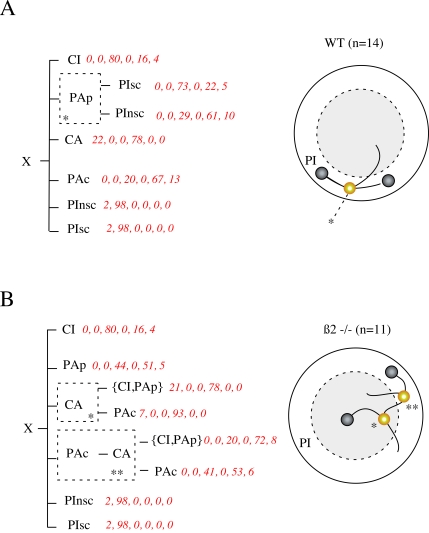
Architecture of sequences using Variable Length Markov chain formalism. Sequences are described using 6 states CI, PAp, CA, Pac, defined as previously, and PInsc and PIsc that correspond to PI without or with scanning. Context tree is drawn in landscape mode with the root (X) placed on the left and past dependencies on the right. Probability distribution over the next symbols appears after each context in red (percentages). For example for WT, (0,0,80,0,16,4) indicates that P(X|CI) = 0; 0; 80; 0; 16 and 4% for X = CI , PAp, CA, PAr, Pinsc and PIsc respectively. Each horizontal line indicates a step in the past. {} indicates a choice between different symbols (A) Fitted context tree (Left) for concatenated sequence of n = 14 WT animals. and schematic representation (Right) of the “choice point”, to enter or not in the center after a PI (B) Fitted context tree (Left) for concatenated sequence of n = 11 ß2−/− animals and schematic representation of chaining (Right).

The WT mice context tree (Figure 7A, left) showed seven contexts. Five of them were first order (from top to bottom, CI, CA, Pac, PInsc and PIsc, Figure 7A), indicating that the next symbol (X) depends uniquely on the present state. More interestingly, two contexts with second order also appeared. The first corresponded to the previous demonstration that after “scanning” an animal tended to engage more frequently in a transition to the center of the arena. The second indicated that, in contrast, when mice did not perform scanning, they preferentially made a stop in the periphery. This is schematized (Figure 7A, right) by a “PI choice point”, where the movements that follow depend on what activity the mouse had performed during the previous PI.

The context tree of β2−/− mice was made of eight contexts, four of them (CI, Pap, PInSC, PIsc) being of first order. The architecture of the tree was clearly modified when compared to WT. Strikingly, dependence between movements during PI and “transition to center” completely disappeared. In contrast, the tree highlighted different chains in the ß2−/− sequence of behavior, with chains of second or third order that organized movements and relations between PAc and CA (Figure 7B).

## Discussion

In this paper we have investigated the processes underlying ß2−/− mouse hyperactivity in an open field. These mice exhibit an increase in the total distance traveled in the open field by about 40% when compared to WT. Consistent with this hyperactive phenotype, ß2−/− mice spent more time in fast, and less time in slow, movements. To analyze mouse trajectories we developed a specific approach based on a dissection of mouse behavior in the open field as a sequence of motor activities organized in patterns. We have shown evidence for two main modifications of the behavior in ß2−/− mice: (i) quantitatively, mutant mice show a reduced number of stops and modification of specific transition probabilities, and (ii) structurally, the organization of the sequence of behavior was different between strains.

Streams of complex acts or movements exhibit some regularity that is the basis of the subdivision of behaviors into units, or species-specific movements. In rodents, a variety of complex sequences of action have been identified [20]. In our analysis we focused on two classifications, active versus inactive, and central versus peripheral movement. Although simple, this classification captures two essential and ethologically meaningful properties of the displacement. The first is the alternation between progressions and stops, observed in a number of locomotor behaviors, and associated with prey search, vigilance or energy saving [21]–[23]. The second concerns the spatial distribution of movement. Traveling close to the wall is an important feature of the mice, and it has been suggested that the wall confers security while the center is anxiogenic. However, exploratory behaviors also drive the mouse to explore all the open space. A more precise definition of the different movements can be performed [15],[24], but our coarse-grained decomposition allowed us to focus on sequence properties, and to obtain sufficient stationary data in 30 min experiments, for a robust statistical description of simple spontaneous decision making (engage in the center of the arena, stop…).

Analysis of behavior in terms of sequences and Markov processes has been already applied to different species [25]. Markov analysis assumes that the underlying process that generates a sequence is homogeneous in time all along the sequence. The time range over which an event influences the future ones is supposed to be constant (i.e independent of the event and the sequence preceding it). For this reason, fixed length Markov chain analysis is a poor detector of sequence rules that operate only after a particular portion of the sequence. By contrast, VLMC allows identification of particular sequences or contexts, such as those identified after scanning an environment. Modification of this homogeneity in sequences is often seen as an indicator of higher organization such as “hierarchical” or “grammatical” properties [26],[27] or reflects specific ‘decisions’ [26]. The methodology applied in this paper is not intended to be a blind modeling but rather a way of testing hypotheses, giving or not significance to ‘a priori’ choices and categories. It offers the possibility of including ethological knowledge and previously established categories. It would then also be relevant and efficient also in more naturalistic and complex settings. The VLMC framework can be generalized so as to investigate whether the grouping of categories in classes is relevant. It thus proves to be useful to improve the parsimony of the description [28].

Hyperactivity in an open field can take different forms, including faster locomotion, longer periods of travel, fewer pauses, shorter pauses, etc. The question is then whether the reduction of the number of stops is sufficient to explain the hyperactive profile. Our experiments demonstrate that locomotion is not faster in ß2−/− mice, and that the difference lies in the patterns and organization of behaviors. Furthermore, a simulation approach suggests that hyperactivity cannot be explained only by changes in the matrix, or only by changes in the duration of the various states, but by their joint effect. Hyperactivity would then emerge from alterations of many different underlying processes. However, we here propose that in ß2−/− mice hyperactivity is mainly due to the “lack of stops”. Most characteristics of the sequences of ß2−/− mice can be explained by the fact that these mice do not observe certain “stops” and that after a stop they organize their behavior differently. The significance of such a modification and the underlying changes it reflects is, however, not trivial.

**Figure 8:**
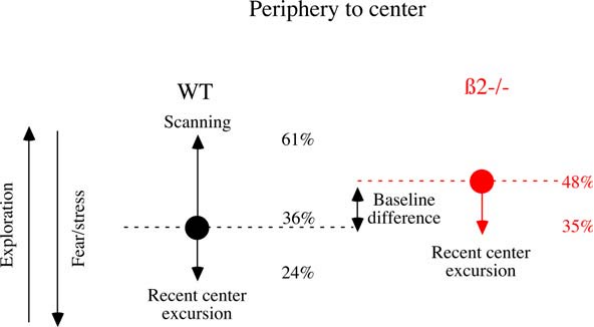
Summarizing context dependent modification of probabilities. Schematic representation of the modulation of the probability to engage a movement in the center of the arena after a stop at the periphery for WT mice (black) and ß2−/− mice (red). Baseline probability (filled circles and dashed lines) is increased (upward arrow) or decreased (downward arrow) by scanning or recent center excursion respectively. Range between the two baselines (dashed horizontal line) marked baseline difference between WT and ß2−/−. Fear and stress (downward left array) are supposed to decrease center excursion while exploration increases (up-ward left array) it.

Open-field behavior, also called exploratory behavior or locomotor behavior in a novel environment has been initially used as an indicator of anxiety/emotionality [10],[11]. It is also used to study exploration and how animal react to novelty, an approach with known limitations [10],[13], the most important difficulty being that the various open-field measurements do not represent a single dimension of behavior (i.e, emotionality or exploration). This limitation reinforces the interest of using sequence analysis, which does not make any assumptions about any underlying process, but focuses on the organization of behavior (see also [29]). Most important features of an animal's displacement organization can be summarized as follows (Figure 8): At the periphery, after a “stop”, the probability that WT mice engage movement in the center of the arena is 36%. This probability is (i) increased by “scanning” (up to 61%) and (ii) decreased by a recent excursion to the center (down to 24%). In ß2−/− mice this probability is different in baseline (48%), the increase caused by scanning disappears and the decrease by recent incursion is similar. These results point to information gathering as a key element underlying differences between WT and ß2 in the organization of sequence of behavior in an open field.

The ability to adapt to an unfamiliar or uncertain environment is fundamental, and an essential point in adaptation would be that animals actively look for a modification in the environment. Displacement of an animal in a novel environment is characterized by intermittent locomotion, scanning, and pauses that can be used to gather information about environment but also to reduce unwanted detection by an organism's predators [22]. Organization of locomotor behavior in an open environment is compatible with optimization theory insofar as it minimizes risk while maximizing gain, i.e. collect information about environment [30]. Fear and anxiety tend to reduce center movement, while exploratory motivation tends to increase these movements [24]. Accordingly, increased probability of center engagement after scanning may be viewed as caused by a reduction of anxiety (Figure 8). Yet, WT and ß2−/− mice have similar levels of anxiety [4],[31], furthermore the parallel evolution of CA → CI probability of transition suggest that reduction of anxiety with time is similar in both strain. The observation that the structure of the displacement is modified in ß2−/− mice and that this modification targets “scanning” as a key feature in the organization of behavior suggests instead a modification of information gathering and of the risk/gain optimization. The notion that exploratory behaviors in novel environments may serve to optimize safety and that this behavior is modified in ß2−/− mice also parallels previous observations suggesting that WT mice react to novelty by increasing exploratory activity, whereas ß2−/− mice do not adapt their behavior to a change in the environment [4].

It has been proposed that the alteration of behavioral adaptation in ß2−/− mice, coupled with unimpaired memory and anxiety, may model cognitive impairment observed in human disorders [4] such as attention-deficit hyperactivity disorder (ADHD) [32], or even in autism [5]. This proposition relies upon the idea that behavioral flexibility is controlled by an adequate hierarchization of motivations, a process known to mobilize prefrontal and cingulate cortex. ADHD symptoms such as inattention lack of inhibitory control, and hyperactivity and prefrontal involvement indeed resemble ß2−/− behavioral deficits, and fit well with nAChR localization and function. Yet, the possible contribution of prefrontal cortex and higher-level top-down processes in open-field behaviors is at this stage not clear. More complex environments and tasks, together with relevant methods of analyses, are needed to explore this problem. Further experiments are also needed to clearly identify the brain loci and the nicotinic receptor subunits that are involved in the modification of the behavioral patterns observed in ß2−/− mice. This fine-tuned analysis of the way wild-type and mutant animals organize their spontaneous activity may ultimately help to understand the contribution of nAChRs to higher brain functions in humans, and the abnormalities that accompany many neuro-pathologies.

## Methods

### Data Acquisition

Exploratory activity was recorded in a 1-m diameter circular open-field. Experiments were performed out of the sight of the experimenter and a video camera, connected to a Videotrack system (View-point, Lyon, France), recorded the trajectory of the mouse for 30 minutes. To characterize stopping behavior ethologically, home-made softwares (Labview, National instrument) were used to acquire film with a higher resolution.

### Symbolic Representation of the Animal Trajectory in an Open-Field

Initially introduced in a purely mathematical context, symbolic dynamics has also been developed as an efficient tool for data analysis [33]. It provides a framework to investigate generic features of a dynamical system from the knowledge of experimental trajectories, in particular when only short series are available, when individual variability is important, or when only a few features within the recording are relevant. The core idea is to encode continuous-valued trajectories into behaviorally relevant symbol sequences associated with a finite partition of the state space. Velocity and position of the mice were used to define a partition in four states (or symbols), by combining two binary ones (see below):

1. An organism's locomotor behavior consists of an alternation between progression and stopping. These alternations have been shown to be ethologically meaningful [14],[21]. In order to capture this property, we partitioned instantaneous velocity values by a threshold into two states A and I corresponding respectively to active priods and inactive or stopping periods (see Figure 1B).
2. Mice in a circular arena travel in both the center and along the perimeter of the open-field. Traveling close to the wall is an important feature of the mice behavior, and it has been suggested that the wall confers security while the center is anxiogenic. However, exploratory behaviors also drive the mouse to explore all the open space. Spatial distribution of mouse position is then expected to be non homogeneous. To account for the spatial organization of the open-field behavior, the arena is then divided into two regions, a central zone C (Centre) and an annulus P (periphery).

When combined, these symbols give four codewords or states *{PA*, *PI*, *CA*, *CI}* that correspond to Activity or Inactivity in the Periphery or in the Center of the arena. Animal trajectories in the open-field are then represented by a sequence of codewords (Figure 1C). The choice of a specific threshold value to partition symbols and the range of validity of these values have been discussed and analyzed in a previous paper (see Supporting Information [6]).

### Symbol Definition: Activity/Inactivity

The 2-D paths were smoothed using triangular filter. The instantaneous velocity can be then meaningfully computed from these smoothed data, simply implementing its definition (first time-derivative of the position).
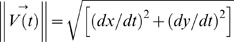



Instantaneous velocity range was partitioned in two sub-ranges delineated by the threshold θ_1_. A second threshold θ_2_ has to be involved in order to faithfully assess activity, according to the following rule:
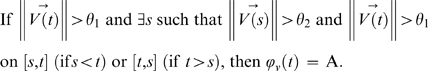
allowing to encode the continuous trajectory into a binary sequence *φ*_v_(t). In other words, it means that crossing the low threshold *θ*_1_ can be considered as the starting point of a significant active phase if and only if the velocity reaches the high threshold *θ*_2_. This high threshold determines qualitatively the active type of the period whereas the low threshold determines quantitatively its duration. This dual criterion avoids spurious alternation of active and inactive phases of arbitrary small duration. Indeed, since the acceleration of the mouse is bounded above by some value a_max_, the duration of an active phase is at least (*θ*_2_-*θ*_1_)/*a_max_* hence the choice of the thresholds implicitly fixed a lowest bound on the time scales. In fact, a lowest bound on the time scale was also prescribed explicitly: an additional temporal smoothing achieving a stronger masking of fast velocity fluctuations is performed by fixing a minimal duration above or below the low threshold to record it as an actual crossing.

The two-threshold criterion masks the presence of weak peaks in the velocity that do not overwhelm significantly *θ*_1_ (even if they last long) while the explicit constraint on duration masks the narrow peaks (fast fluctuations) even if they reach high velocity values. The combination of these two criteria moreover ensures that the resulting binary sequence is not very sensitive to the precise value of *θ*_1_ (this feature has also been checked directly).

### Symbol Definition: Spatial Location, Center/Periphery

The area of the arena was divided in two regions, with a central zone C (Center) with R_c_<1 and an annulus P (periphery). Then, depending on the continuous radial position R(t) = (x^2^+y^2^ )^1/2^, defined in such a way that it ranges from 0 to 1 depending on whether the mouse was close to the border of the arena (*R* = 1) or at its center (*R* = 0), the trajectory of the mouse is transformed into binary sequence *φ*_p_(t) by:
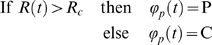



In this study Rc = 0.65.

### Ethological Classification

In order to be able to differentiate patterns of inactivity, video of the animal displacement was recorded (25 frames/second) and used to detect the position of the animal. To classify the stops without bias, only parts of the movie considered as PI in the behavioral sequence were watched without looking either at the duration of the stops, or at the following sequence. We used five classes of behavior for this classification, rearing, grooming, border rearing, sniffing and scanning [20]. Such an ethological classification has been chosen for its clarity as regarded the aims of the different behaviors. Grooming is defined by a well-characterized sequence beginning with movements of paw cleaning and proceeding through face washing and body cleaning. Rearing and border rearing were easy to distinguish, the animal raises upon its back paw. Difference in between rearing and border rearing is whether front paw touch the border of the open-field or not. Sniffing is defined by an activity in which the mouse sniffs the ground, this behavior is usually used to identify object or food or to make spatial landmark. Scanning contains any information gathering about the environment, beginning with rearing but the animal then engages large head movement that can be accompanied by sniffing.

### Matrix of Transition and Flow Diagram

Henceforth, we shall call “symbol” each of the 4 codewords PA, PI, CA, CI since the binary symbols will never be considered in isolation in what follows.

One way to analyze a sequence consists in analyzing the probability of transition from one state to another. From the initial time series written with an alphabet of x symbol, a x*x matrix T = (tij) can be calculated, where tij is the number of times a given symbol i is followed by another symbol j in the sequence. T is called a transition frequency matrix. A conditional transition matrix can be obtained by dividing each row of the transition frequency matrix by its sum. Conditional probabilities for each state are then estimated by unbiased estimator p(A|B) = n(BA)/n(B) where (n(BA) designates the number of 2 symbol sub-sequences where B is followed by A. Transition frequency matrices and conditional transition matrices are a concise way of expressing the statistical relationship between consecutive states. They give preliminary clues to the organization of the sequence of states. This is generally summarized in a flow diagram, giving a simple graphical representation of these matrices. Nodes in the diagram represent states, while arrows of variable thickness represent the frequencies with which the different transitions occur. This representation provides a suitable overview of the organization of the sequence of behaviors (see Figure 3).

### Markov Chain

The matrix of transition describes the statistics of transitions from one state to the other but it does not provide any information about the dynamic nature of the relationship between successive states. Obtaining information about the dynamics in short and long terms from the sole knowledge of the transition matrix is possible only if the dynamics is Markovian: A process is a first-order Markov chain if the transition probability from state A to the next state B depends only on the present state A and not on the previous ones. A first-order Markov model is then a mathematical model fully prescribed by the transition matrix that describes, in probabilistic terms, the dynamic behavior of the system, namely the probability of transitions over any duration between any two states. In such a model, the present state contains all the information that could influence the choice of the next state, that is captured in the transition matrix. A classical way to demonstrate that a process is Markovian is to show that the sequence cannot be described by a zero order process, i.e. that P(B|A)≠ P(B) and that P(C|B) = P(C|AB), but see [25] for a more detailed review of all these methods.

The residence times, defined as the time spent in a given state, were studied separately. We described the dynamics of transition between states using an alternate renewal process. That is the sequence is described by the convolution of a Markov chain describing the transitions between the states associating a unit time step to each transition, with the above residence-time distributions, describing the actual duration of each step. Thus, there is no repetition of states in the sequence and the transition matrix has vanishing diagonal elements

### Modeling Strategy

The most interesting part of the Markov formalism is that the knowledge about the transition probability, i.e. the elementary properties of the system, is sufficient to describe the whole dynamics of the system, either in the short or long term. In practice, this means that as soon as a first-order Markov process has been demonstrated, modifications induced by drugs, genetic mutation or other manipulation of the system can be localized in the transition probabilities and/or in the time distribution of state duration (provided the investigated perturbation does not affect the first-order nature of the dynamics) and the same modeling strategy can be used.

Modeling procedure is as follows. We used (i) the conditional probabilities from a given state to specify the next one, and (ii) the residence time distributions to determine durations of the successive states. This whole procedure is reiterated until the total duration reaches half an hour of experiment. These synthetic data can then be compared with those obtained experimentally. In a second time, specific modification of transition probabilities or residence time distributions are used to access impact of such a modification.

A specific model, consisting in “stop reduction” has been particularly used. In this model, sequences of symbols are generated using WT matrices and distribution. In a second step a fixed percentage of stops (35% of both PI and CI) are removed in such a way that PA-PI-PA becomes PA-PA, that is a unique PA event but with a longer duration (and similarly for CA-CI-CA). The total length of the sequences is adjusted in a way that it represents a half-an-hour experiment.

### Variable Length Markov Chain Analysis

When the dynamics is not accounted for by a first-order Markov chain, but displays larger dependence on the past states, “variable length Markov chains” (VLMC) provide an efficient modeling [19]. In this class of models, dynamics is still prescribed by the expression of conditional probabilities of the future states. But now, each history from *t* = −*∞* up to time *t* is truncated into finite sequence from *t-s* to *t*, with s≥0, having actually an influence onto the states at time t+1. For all B, P(B at *t*+1 | past up to t) = P(B at t+1 | C(past, t)). The length of the truncated sequence C (past, t), called a context, depends on the history instead of being uniformly equal to the length of the longest one. The gain in reducing the dimension of the parameter space is obvious when the dynamic memory is heterogeneous (context-dependent).

A VLMC is thus characterized by: (i) a set of finite-length context, and (ii) a family of transition probabilities associated to each context. The context defines the finite portion of the past that is relevant to predict the next symbol (whatever it is). Given a context, its associated transition probabilities define the distribution of occurrence of the next symbol.

VLMC analyses were performed on concatenated chains obtained from different animals of the same group. The R-package VLMC was used to fit data. Fittings were performed in two steps. First a large Markov chain is generated containing the context states of the time series. In our analysis only nodes that appear n = 5 times per animals (that is 70 for 14 WT and 55 for 11 β2−/−) were taken into account to generate the initial tree. The obtained results are almost insensitive to the value of this parameter n. In the second step, many states of the Markov chains were collapsed by pruning the corresponding context tree. The pruning requires definition of a cutoff value. A large cutoff yields a smaller estimated context tree. In our analysis cutoff value corresponding to 1‰ was used in order to extract strong and significant contexts.

### Statistical Analysis

All data were analyzed using R, a language and environment for statistical computing. Data are plotted as mean±95% confidence intervals. Boxplot is also used when information about distribution is important (see Figure 2A and 2B, for example). Boxplot summarizes data using the smallest observation, lower quartile (base of rectangle), median (line in rectangle), upper quartile (summit of rectangle), and largest observation. Data points considered outliers are marked by isolated points (circle).

Total number (n) of observations in each group and statistics used are indicated in figure captions. Classically comparisons between two means are performed using two-sample t.test. When there is doubt about the normality of the data distribution, non-parametric Wilcoxon rank-sum test is preferred. For variable Markov chain model fitting, VLMC package is used.

## Supporting Information

Figure S1 Comparison of simulations using Markov, semi-Markov and non-stationnary models (see Text S1) (A,B) Simulation of the time spent in PI, CI, PAc, CA and PAp states (from left to right) using different models. No clear cuts were observed when comparing (A) Markov (circle) and semi-Markov models (triangle) and (B) Markov (circle) and non-stationary Markov models (triangle) (C,D) Simulated time spent in PI (left) and PAc (right) obtained by combining transition matrices and distributions of state durations. WT/WT, ß2/WT, WT/ß2 and ß2/ß2 indicate that sequences are simulated using WT or ß2−/− matrices of transition (before /) and WT or ß2−/− state duration distributions (after /). (e.g., WT/ß2 indicates simulation with WT matrix of transition and ß2−/− residence time distribution). "Matrix" and "Time" indicate that the discrepancy originates from the effect of changing the transition matrix and the residence time distribution, respectively. (C) Comparison between Markov (circle) and semi-Markov models (triangle). (D) Comparison between Markov (circle) and non-stationary Markov models (triangle).
(1.27 MB TIF)

Figure S2 Simulation of the sequence. (A) Comparison between the number of PI, CI, PAP, CA and PAc states (from left to right) in WT (black circle), ß2−/− (red circles), simulation obtained from WT first-order transition matrix and residence time distributions (black triangle) and simulation obtained from ß2−/− first-order transition matrix and residence time distributions (red triangles). Note that distributions of experimental and simulated data fit perfectly. (B) Typical recurrence plot of an experimental sequence (left) and a simulated sequence, in WT (B1) and in ß2−/− mice (B2).
(7.35 MB TIF)

Text S1 Supplementary material file and legends for Figure S1 and Figure S2
(0.04 MB DOC)
