# Supplementary figures and images for: Behavioral Sequence Analysis Reveals a Novel Role for ß2* Nicotinic Receptors in Exploration
Source: PLoS Comput Biol. 2008 Nov 21;4(11):e1000229. doi: 10.1371/journal.pcbi.1000229 (PMC2581917; doi:10.1371/journal.pcbi.1000229)

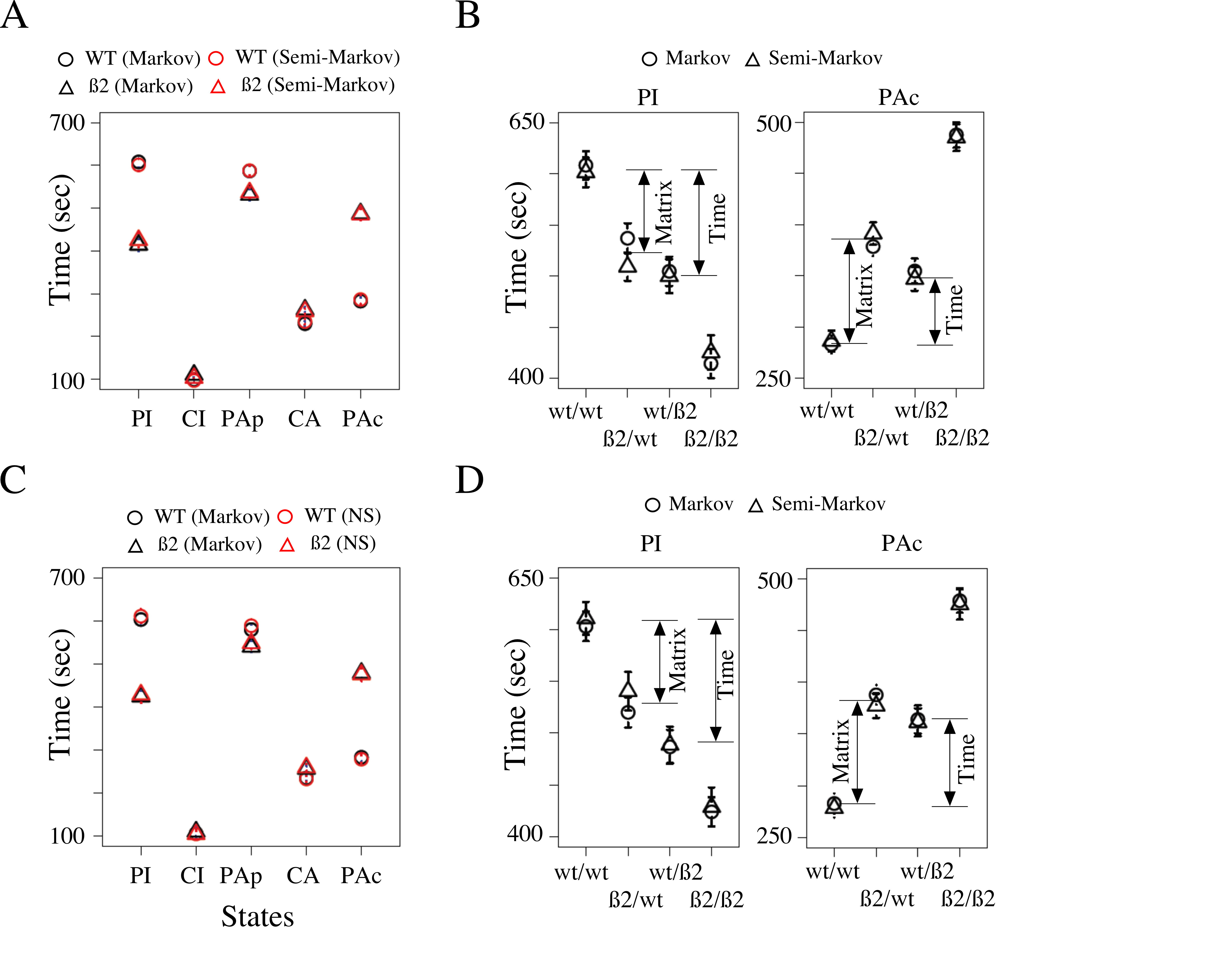

Supplement: Figure S1 — Comparison of simulations using Markov, semi-Markov and non-stationnary models (see Text S1) (A,B) Simulation of the time spent in PI, CI, PAc, CA and PAp states (from left to right) using different models. No clear cuts were observed when comparing (A) Markov (circle) and semi-Markov models (triangle) and (B) Markov (circle) and non-stationary Markov models (triangle) (C,D) Simulated time spent in PI (left) and PAc (right) obtained by combining transition matrices and distributions of state durations. WT/WT, ß2/WT, WT/ß2 and ß2/ß2 indicate that sequences are simulated using WT or ß2−/− matrices of transition (before /) and WT or ß2−/− state duration distributions (after /). (e.g., WT/ß2 indicates simulation with WT matrix of transition and ß2−/− residence time distribution). "Matrix" and "Time" indicate that the discrepancy originates from the effect of changing the transition matrix and the residence time distribution, respectively. (C) Comparison between Markov (circle) and semi-Markov models (triangle). (D) Comparison between Markov (circle) and non-stationary Markov models (triangle). (1.27 MB TIF) [file pcbi.1000229.s001.tif]
