# Supplementary material for: Behavioral Sequence Analysis Reveals a Novel Role for ß2* Nicotinic Receptors in Exploration
Source: PLoS Comput Biol. 2008 Nov 21;4(11):e1000229. doi: 10.1371/journal.pcbi.1000229 (PMC2581917; doi:10.1371/journal.pcbi.1000229)
Supplement: Text S1 — Supplementary material file and legends for Figure S1 and Figure S2 (0.04 MB DOC) [file pcbi.1000229.s003.doc]

# Supplementary material

Validity and limit of the first order Markov model

A First Order Markov model and simulations of time spent in a given state suppose i) that the sequence of states is stationnary, and ii) that the resident time within a given state does not depend on the previous one. Both assumptions have shown to be partially false (see text). We here show the impact of such properties on the simulation of the time spent in a given state. This is important in order to identify key elements that could explain differences between WT and ß2-/- mice.

In a first series of simulations, the impact of residence time description is quantified by comparing in the WT and ß2-/- a Markov model. Thus, residence time is described by five distributions estimated from experimental residence time within each of the five states and a semi-Markov model, i.e. residence time is described by 9 distributions corresponding to residence time in Pap, Pac and CI, resident time in CA preceed by Pap, Pac or CI, and resident time in PI preceed by Pap, Pac or PI. Similarly, the impact of non-stationarity is made by comparing the simulations of time spent within a given state using i) two matrices obtained from sequence in WT and ß2, and ii) four matrices obtained from sequences in WT and ß2 during the first and the second 15 minutes.

These simulations showed that a semi-Markov model, or a Markov model with non-stationarity, have little impact on the time spent in a given state estimation when compared with a Markov model (Fig S1 A, C). Furthermore, the role of matrix versus resident time is similar in all simulations (Fig S1 B, D). This suggests that non-stationarity and variation of time spent in a given state and its dependence on the preceding state are important biological information. But they cannot explain the difference between WT and ß2-/-. It should be clear, however, that the Markov Model cannot be used to account for adaption such as the modification of transition probability (see text). To do so, a modification of the transition matrix with time should be implemented.

We also verified that the numbers of each of the five states in the 30min experiment agree well in WT and ß2-/- mice with simulated data when the respective matrix of transition and residence time distribution are used (Fig S2 A). The temporal structure of a chain can be described in recurrence plots [34,35]. Such plots that identify the structure of recurrences along the chain of symbols show a number of checkerboard structures in WT mice (Figure S2 B1 Left). Such structures correspond mainly to PI-PA-PI sequences and evidence typical behaviors in WT animals consisting of alternating movement at the periphery intermingled with sequences of center movement. In ß2 -/- mice such checkerboard structures disappear (Figure S2 B2 Left). Differences between transition probabilities within a first-order Markov Model are sufficient to explain this difference in recurrence plot structure as illustrated on two recurrence plot of WT (Figure 4B1 right) and ß2 (Figure 4B1 Left) modeled data. Calculation of various indices devised to quantify the recurrent patterns in recurrence plots [34,35] and of Lempel-Zev complexity [36] demonstrate that the temporal organization of the sequences of symbols is well explained by the first-order transitions between states.

34. Faure P, Korn H (1998) A new method to estimate the Kolmogorov entropy from recurrence plots: its application to neuronal signals. Physica D 122: 265-279.

35. Marwan N, Romano M, Thiel M, Kurths J (2007) Recurrence Plots for the Analysis of Complex Systems. Physics Reports 438: 237-329.

36. Rapp P, Watanabe T, Faure P, Cellucci C (2002) Nonlinear signal classification. International Journal of Bifurcation and Chaos 12: 1273-1293.

**Figure S1: Comparison of Simulation using Markov, semi-markov and non-Stationnary model (see text suppelemntary material) (A-B) Simulation of the time spent in states PI, CI, PAc, CA and PAp states (from left to right) using different models.** No clear cut were observed when comparing (A) Markov (circle) and semi-Markov model (Triangle) and (B) Markov (circle) and non stationary Markov model (Triangle) **(C-D)** **Simulated time spent in PI (left) and PAc (right) obtained by combining transition matrices and distributions of state durations.** WT/WT, ß2/WT, WT/ß2 and ß2/ß2 indicate that sequences are simulated using WT or ß2-/- matrices of transition (before /) and WT or ß2-/- state duration distributions (after /). (e.g. WT/ß2 indicates simulation with WT matrix of transition and ß2-/- residence time distribution). “Matrix” and “Time” indicate that the discrepancy originates from the effect of changing the transition matrix and the residence time distribution respectively. (C) Comparison between Markov (circle) and semi-Markov model (Triangle). (D) Comparison between Markov (circle) and non-stationary Markov model (Triangle).

**Figure S2: Simulation of the sequence. (A) Comparison between the number of PI, CI, PAP, CA and PAc states** (from left to right) in WT (black circle), ß2-/- (red circles), simulation obtained from WT first-order transition matrix and residence time distributions (black triangle) and simulation obtained from ß2-/- first-order transition matrix and residence time distributions (red triangles). Note that distributions of experimental and simulated data fit perfectly. **(B) Typical recurrence plot** of an experimental sequence (left) and a simulated sequence, in WT (B1) and in ß2-/- mice (B2).
